# Everyday Activities of Older Adults in Tianjin, China: Coupling Constraints, Gendered Mobilities and Social Context

**DOI:** 10.3390/bs13120996

**Published:** 2023-12-04

**Authors:** Yaqian Mao, Tomoko Kubo

**Affiliations:** College of Life and Environmental Sciences, University of Tsukuba, Tsukuba 3058572, Japan; s2130283@u.tsukuba.ac.jp

**Keywords:** behavioral geography, everyday life, older adults, coupling constraints, gendered mobilities

## Abstract

Many Western studies have indicated that older women are generally more vulnerable in terms of mobility compared to older men, particularly regarding driving. However, the situation may differ in the context of China. This study, based on activity diaries and semi-structured interviews, focuses on the spatiotemporal behavior of older adults in Tianjin and explores how the constraints posed by activity companions (in terms of type, size, and composition) shape the mobilities of older men and women, including activity locations, travel distances, and transportation modes. The key findings are as follows: First, older women are more engaged with their families due to a higher percentage and longer duration of activities spent with family members. Second, older men tend to have more concentrated travel distances near their homes compared to older women. Third, older women exhibit a broader range of activities in different locations and engage in longer-distance leisure travel with family members when compared to older men. In the context of Western literature, this study discusses older women’s enhanced social interactions, their earlier retirement in China, and the impact of COVID-19 as factors that help explain these findings. This study contributes to a deeper understanding of accompanied mobilities among Chinese older adults using geographical theory and methods, emphasizing the importance of flexible work schedules for the workforce and the organization of community-based activities to promote the social interactions and mobilities of older adults.

## 1. Introduction

Maintaining mobility in aging cities is one of the most discussed topics in relation to population aging [1,2]. Geographers have shown increased interest in the interrelationship between the mobility of older adults and their well-being, quality of life [3,4,5], planning and policy on aging [6,7], health and place [8], and more. Numerous individual and contextual factors influence the mobility characteristics of older adults, such as household structure and social networks [9], life cycle [10], racial effects [11], age-related disabilities [12], cohort effects [13], accessibility of transport services [11], and gender.

While there is a growing body of literature discussing the mobility of working younger women, there is relatively less research on retired older women. Gender-related research on mobility aims to understand gender as a relational category with implications for both men and women [14,15,16,17]. Hjorthol, Levin, and Sirén [13] examined the travel and activity patterns of older people and found that older women are more vulnerable than older men in terms of holding licenses, access to cars, and driving experience. Nordbakke [18] studied the barriers, strategies, and options faced by older women in mobility through interview analysis. She provided evidence that older women face difficulties in accessing cars and driving, emphasizing the importance of a social network in promoting their mobility. While most geographers have sought to improve older adults’ mobility by analyzing built-environment factors such as land use features and transportation services [19,20], these two studies offer a gendered perspective on understanding how gender shapes the mobility of older women from a behavioral perspective, especially concerning driving mobilities. Nordbakke’s study emphasizes the significance of social networks in fostering the mobility of older women.

However, their findings may not be directly applicable to the Chinese context, and further exploration is warranted regarding how social networks or activity companions shape older adults’ mobilities. Topics such as low mobility caused by the lack of a driving license among older adults have not attracted much attention in China’s context [21], due to variations in social factors such as retirement age, household structures, diverse living habits, and preferred modes of transportation [22,23]. Walking and cycling are the most frequently used transportation modes in China’s context [24].

In addition, few studies have delved into the gender aspects of Chinese older adults’ mobility. Feng et al. [24] explored gender issues in relation to older adults’ mobilities, taking into account household size and emphasizing the roles of gender and social networks. However, similar to previous literature, the features of activity companions are not mentioned and require further exploration.

An activity companion is a vital factor that significantly influences individuals’ behavior. It is particularly crucial to study retired older adults, who are in the last stage of their life cycle and often seek companionship and social support. These companions can be considered as components of social networks [25,26,27,28], sources of social capital [29], and providers of social support [30]. These aspects are essential for enabling individuals to live within society and establish meaningful connections with others. The concept of coupling constraints, originating from the theoretical framework of time geography developed by Hägerstrand [31], pertains to the determination of when, where, and for how long individuals (both human and non-human) are physically present. Consequently, this concept is frequently used to structure the everyday activity and travel patterns of individuals from a spatiotemporal perspective [32,33]. This approach contributes significantly to our understanding of how various types of households manage the balance between work and domestic responsibilities [34]. In a study by Frantál, Klapka, and Nováková [35], the impact of companions on individuals’ lives was examined by measuring the frequency of interactions with companions, with a positive outcome in terms of reducing isolation. Another study by Mao and Li [9] analyzed activity diary data, revealing a tendency for travel distances to decrease with fewer family members. While these studies have contributed to our understanding of the impact of companions on older adults’ daily mobility, they did not clearly define the size, type, or composition of companions, nor did they specify the temporal characteristics of these activities.

China is confronted with the challenge of having the world’s largest elderly population, with over 200 million individuals aged 65 and above in 2021 [36]. This demographic reality has presented significant societal challenges, including an increasing burden of caregiving responsibilities on families and the government, an imbalanced population structure across different provinces, a labor shortage, and impediments to economic development [37]. The city of Tianjin, situated adjacent to Beijing, holds immense political and economic significance for both the capital and the nation. With a registered population of 11.51 million, Tianjin grapples with demographic statistics that reveal 32.25% of its population being aged over 60, and 22.97% aged over 65 [38,39]. In terms of family structure, 21.38% of individuals over 65 years old in Tianjin live alone [38]. This prevailing demographic challenge, along with the associated social interactions among older adults, raises concerns. Existing literature suggests that limited social interactions may result in poorer mobilities among older adults. Therefore, it is imperative to gain insights into the mobility patterns of older adults in the specific context of Tianjin and, by extension, in China.

Based on the review of the existing literature, it is evident that there is a significant research gap concerning gendered mobility among older adults within the specific context of China. Furthermore, the contribution of activity companions on the mobility of older adults has not been adequately explored. The significance of Tianjin, given its national importance and the substantial proportion of the aging population within its boundaries, cannot be overlooked. Consequently, this study seeks to address the following questions within the context of Tianjin, China: When and where do older women and men engage in their daily activities? What is the duration of their travels and what are their preferred modes of transportation? What are the size, type, and composition of their activity companions? How do these characteristics of activity companions influence their choice of activity locations, travel distances, and transportation modes? To gain insight into the complex interplay between the attributes of companions and the mobility patterns of older adults, with a focus on gender differences, this study employs diary surveys and semi-structured interviews to explore how coupling constraints shape the daily activities of older adults. The subsequent section details the data collection methods and procedures, followed by a presentation of our analysis of the behavioral data in the third section. Lastly, we delve into the implications arising from our findings within the social context of the study area.

## 2. Materials and Methods

### 2.1. Study Area

This study focused on three parks located in the Binhai New Area of Tianjin, China. Tianjin is a municipality and a major coastal city in Northern China, directly governed by the central Chinese government. The registered population of Tianjin experienced a significant increase from 9.96 million at the end of 2011 to 11.51 million in 2021. During this period, the proportion of the population aged 60 and over rose from 22.77% to 32.25%, and the proportion of those aged 65 and over increased from 16.22% to 22.97% [38,39]. As a sub-provincial area, the Binhai New Area is integral to the national development strategy. Within this district, the population aged 60 and over constitutes 17.15% of the total 2.07 million residents, while the population aged 65 and over accounts for 11.63% [40]. Notably, the Binhai New Area serves as a model region for the development of age-friendly initiatives in Tianjin [41].

Parks are a crucial public leisure resource for older adults in China, serving as spaces where they invest a significant portion of their time, partake in social interactions, and develop their individual identities. Our research took place in September 2021, a period characterized by China’s rigorous anti-epidemic measures, which restricted access to locations outside of individuals’ homes and indoor recreational venues for older adults. These conditions also extended to our team of investigators. Given this context, the significance of parks as places for older adults to pass their leisure time grew exponentially. Consequently, parks became the primary outdoor venues for us to engage in valuable discussions with older adults. Acknowledging that older adults represent a vulnerable group with regard to COVID-19, our investigators strictly adhered to epidemic prevention protocols, and each survey session with a single participant was limited to approximately 15 min.

**Figure 1:**
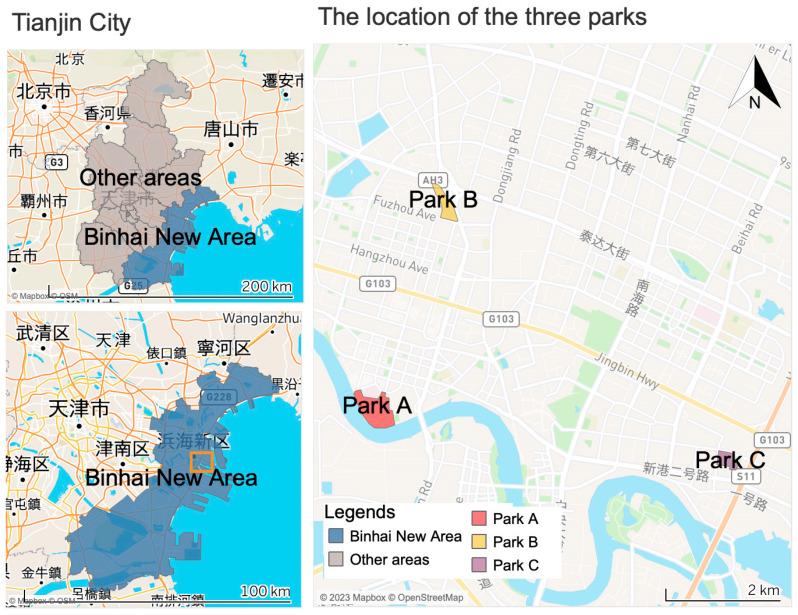
The study area in Tianjin City, China.

The selection of the three parks was based on their location and our fieldwork, as indicated in Figure 1. First and foremost, these parks are strategically situated in the heart of the economic development zone of the Binhai New Area. Second, we conducted extensive surveys covering all the green spaces marked on the map, utilizing both internet resources and on-site fieldwork. Our research revealed that the only green spaces experiencing substantial foot traffic and offering a diverse range of recreational facilities were the three selected parks. In contrast, the other green areas were predominantly characterized by golf courses, ecological reserves, street greening projects, or small parks with limited footfall and a lack of recreational amenities. Third, the three chosen parks are in close proximity to residential neighborhoods and essential facilities, making them exceptionally attractive to local residents, including older adults, seeking opportunities for recreational visits. In more detail, Park X stands out as the largest comprehensive park in the Binhai New Area. It enjoys the advantage of being in proximity to a renowned hospital, a substantial stadium, and a bridge connecting the north and south banks of the Haihe River, all of which are easily accessible on foot. Park Y is strategically located near a crucial overpass, offering convenient access to commercial facilities and governmental offices in all directions. Additionally, it is within easy reach of nearby swimming pools and schools. Park Z is situated adjacent to a hospital, with a prominent main road to the east. In comparison to the other two parks, Park Z enjoys its location near the sea, and its surroundings include numerous state-owned enterprises associated with water transportation.

### 2.2. Data and Methods

The data were collected through a combination of activity diaries and semi-structured interviews. The activity diary, employed as a time–geographic method, allowed us to gather information on individuals’ activities occurring continuously over a defined timeframe. This period encompassed both a typical weekend (Sunday) and a standard weekday (Monday), totaling 48 h. The data collected included details such as the start and end times of each activity, the duration of the activity, the type of activity, the category of activity place, the activity companion, travel distance, travel duration, and the mode of transportation employed. This comprehensive approach encompassed all aspects of older adults’ daily lives, subsistence activities (such as meals and sleep), maintenance activities (including housework and household responsibilities), and discretionary activities (such as recreational and leisure activities) [42]. Activity places were classified into four distinct categories: home, leisure places, service places, and other places. Additionally, the companions involved in these activities were categorized as either family members (spouse, children, grandchildren, children-in-law, and other families) or non-family members (friends, neighbors, coworkers, classmates, and other non-families).

Interviews have been widely recommended as a valuable complement to time-use data, contributing to a more comprehensive understanding of individuals’ behaviors [43]. To this end, we conducted semi-structured interviews concurrently to gain insights into the underlying motivations that guided the participants in their daily routines. These interviews featured inquiries such as “Why do you choose to spend your Sundays with your family?” or “What influences your decision to stroll along the street rather than selecting other locations?” and “What drives you to opt for bicycling as your mode of transportation?” It is essential to highlight that the survey participants, who willingly participated in the study, exhibited a willingness to engage in dialogue and readily shared preliminary insights into the motivations underpinning their activities before the formal interview. These preliminary insights proved to be valuable interview data. Furthermore, the investigators actively guided the participants during the interviews, encouraging them to provide detailed responses regarding the motivations behind their activities.

In September 2021, our survey was carried out through face-to-face interviews by a proficient team of six investigators. This team consisted of three male and three female graduate students, all specializing in social sciences. The investigators were organized into three pairs, each comprising one male and one female investigator, and assigned to investigate different parks. To optimize efficiency and minimize the likelihood of interviewing the same individuals, each pair entered the park through separate entrances and randomly approached older adults for participation. Following an initial explanation of the survey’s purpose and addressing any privacy concerns, the investigators commenced their inquiries. While older adults shared their daily routines, the investigators diligently recorded the information in their diaries. Moreover, the survey was equipped to accommodate dual interviews when necessary. For example, when surveying a couple, the investigators recognized that their daily routines often displayed minimal disparities. Thus, after surveying one member of the couple, the investigators efficiently documented any variances and gathered supplementary information from the other individual. Notably, the text content of semi-structured interviews was meticulously documented by the investigators.

We gathered a total of 218 valid participants, accumulating a comprehensive dataset of 6237 activity records, each accompanied by interview records. The gender distribution was suitably balanced, with 55.50% (*n* = 121) being men and 44.50% (*n* = 97) being women. The participants’ mean age was 68.98 years, ranging from 55 to 69. Notably, we extended the age range to encompass women over the age of 55 and men over the age of 60, aligning with the statutory retirement age in China, including Tianjin, for this generation [44]. Roughly half of the participants resided in two-person households, while a minority lived with someone other than their spouse, such as their child, child-in-law, or another individual. A significant proportion of the participants were engaged in occupations related to production, distribution, transportation, sales, or services. We made deliberate efforts to maintain a balanced representation of participants across the three selected parks. Our data analysis approach commenced with coding and statistical analysis of the diaries to gain insights into behavioral patterns. Subsequently, guided by the interview content, we conducted a deeper exploration of how companions shape the mobility patterns of older adults.

## 3. Findings

### 3.1. Older Women: More Engaed in Family

The majority of activities were carried out alone, accounting for over 50.00% of the total, or in the company of their spouse, representing approximately 30.00%. Notably, participants exhibited a higher likelihood of being accompanied by more than two companions on Sundays compared to Mondays, particularly among older women (Table 1). In the presence of a family companion, older women were actively engaged in activities, conducting approximately six times as many with their adult children, children-in-law, and grandchildren when contrasted with their male counterparts, on both days. Furthermore, the involvement of each family member was more pronounced for older women than for older men.

Only a few types of social compositions included non-family members, with a single non-family companion being the most common composition, as opposed to having two non-family members or a combination of family and non-family companions. Activities that involved non-family members were primarily scheduled for Sundays. However, there were exceptions. In one instance, during our interview with an older woman who was enjoying a leisurely walk with her friend in Park X on a Monday, she expressed, “Weekdays are my free time to spend with my friends! You have no idea how busy I am on weekends when I have to prepare meals for my kids and grandchildren!”

Similar to the participant mentioned above, approximately 17.89% of the participants mentioned that they took on the responsibility of cooking for their children and grandchildren on Sundays, particularly when their family members came to visit. This tradition of family gatherings was one of the reasons they spent time with their families on Sundays. In addition, some participants chose to go sightseeing or visit relatives who lived at a distance on Sundays, often accompanied by their adult children.

Not only do the number of activity records highlight the significance of family interactions on Sundays, but also the duration of activities with various companions, particularly for women. The median activity duration spent alone or with their spouse was 6 to 8 h. The duration of activities involving grandchildren, children-in-law, and children was notably longer on Sundays compared to Mondays. However, exceptions arose when school-aged grandchildren resided with them during the week, with their adult children arranging for the children to visit on weekends. Time spent with children-in-law, as well as the opportunities for interaction, decreased on Mondays. The duration of activities with non-family members remained consistent for both genders on both days. A gender difference was observed, as older men spent 2.83 h with their grandchildren on Sundays and only 0.96 h on Mondays. In contrast, older women spent 6.50 h and 4.67 h with their grandchildren on Sundays and Mondays, respectively. For older men, there were no opportunities for activities with their children-in-law on Mondays, whereas older women maintained a consistent duration of over 3.00 h on both days.

### 3.2. Older Men: Restriced Travel Nearby Home

Home was the predominant activity location for older adults, closely followed by leisure places (including parks, plazas, chess rooms, and neighborhood gardens), as indicated in Table 2. A relatively small percentage of activities occurred in service places (such as supermarkets, health centers, banks, and libraries) and other places (such as family members’ workplaces or schools, other family members’ residences, and streets).

For both older men and women, approximately half of the activities performed at home were subsistence activities. Notably, older men participated in more leisure activities compared to older women on both days (as detailed in Table 2). In contrast, older women were more involved in activities conducted at home. Among the prevalent activities conducted at home, watching television and performing household chores were the most common. Furthermore, the stringent anti-epidemic measures in China restricted the capacity of older adults to partake in outdoor leisure activities during the study period. Many participants expressed apprehensions about the epidemic and exhibited a reduced inclination to visit crowded leisure places.

In the category of “other places”, the streets emerged as important for older men to pass their time. On both Sunday and Monday, a substantial portion of activities in this category, accounting for 34.85% and 38.58%, respectively, occurred on the streets. During our interviews with older men who spent a considerable amount of time wandering the streets, they expressed sentiments such as, “Well, there’s not much to do, so I simply stroll around on the street.” It became evident that these older men frequently participated in solitary activities when compared to other participants. One of them disclosed that he lived alone, leading us to conclude that the absence of companions likely influenced their preference for solitary pursuits.

**Figure 2:**
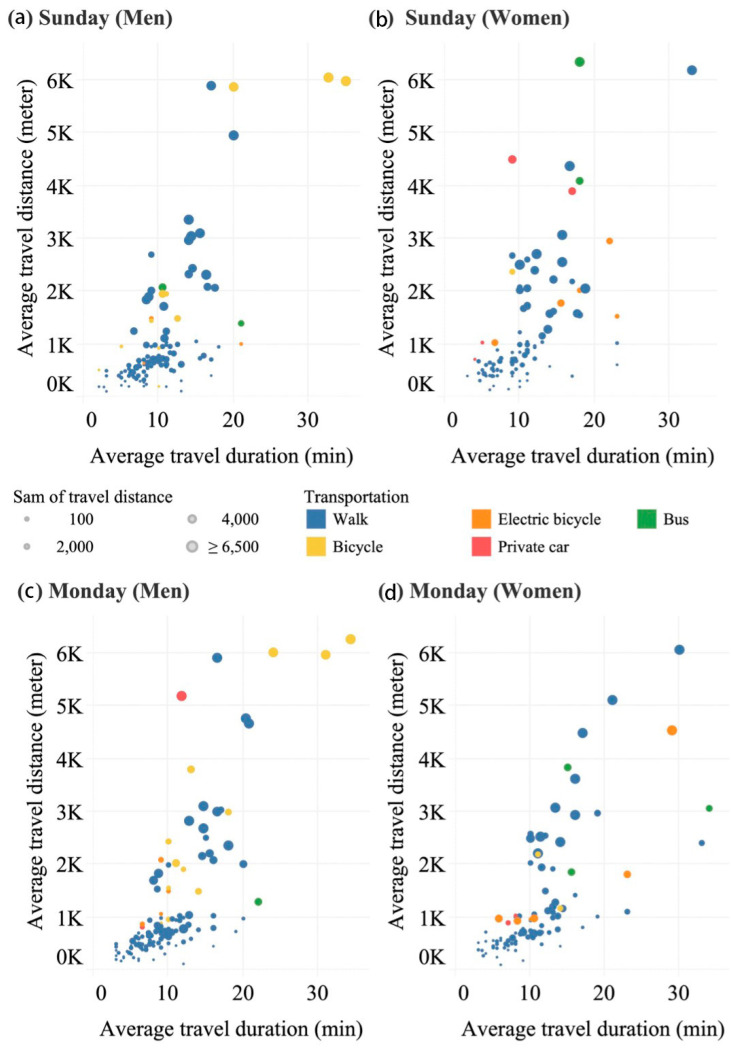
Average distance and duration of travel by various modes of transport on Sunday and Monday for older men and women.

Most activities occurred within a 1500 m radius of the participants’ residences on both Sunday and Monday (Figure 2). After computing the average travel distance and duration, excluding non-daily activities such as sightseeing in other cities, we observed that daily activities typically spanned a radius of 6500 m. On Sunday, 80.00% of travel for older men and 67.80% of travel for older women was contained within a 1500 m radius of home, as depicted in Figure 2a,b. Travel distances surpassing the 1500 m threshold exhibited a more uniform distribution among older women when compared to older men.

The primary mode of transportation was walking, with older women exhibiting a wider range of transportation options compared to older men, as indicated by the comparison between Figure 2b and Figure 2a. However, both genders primarily relied on walking or bicycles for their daily travel, and the difference between the two days was not substantial.

### 3.3. The Power of Older Women’s Companions: Diverse Activities and Travel

This section delves into how the type, size, and composition of activity companions shape the activity location, travel distance, and transportation modes of older adults. Moreover, it elucidates the distinct trends observed on Monday and Sunday.

When examining the distribution of time across various places, it is evident that, with the exception of service places on Monday, older women allocated a higher proportion of their time to all locations when accompanied by family members compared to older men on both days. A particular note is the difference in time allocation to leisure places, where older women engaged in 25.96% of their activities, with 24.5% dedicated to interactions with their grandchildren on Monday. This is in stark contrast to older men, who spent only 0.26% of their time at leisure places on Monday. During the interviews, it became apparent that older women often take on the primary role of caring for their non-school-age grandchildren on weekdays, especially when both their adult children and children-in-law are employed. Furthermore, activities related to caring for their grandchildren extended beyond their homes; they also occurred at leisure locations, where women engaged in playtime with their grandchildren. Thus, factors such as the age of their grandchildren, the employment status of their adult children’s families, and the caregiving arrangements for their grandchildren may contribute to the higher percentage of older women spending time with their grandchildren on Monday.

When considering the travel distance for daily activities within the range of 6500 m for each transportation mode, it is noticeable that older women tended to travel farther than older men when going alone to leisure places, service places, and other places with one family member on Sunday, with differences exceeding 3000 m. On Monday, they also tended to travel over 2000 m when accompanied by a non-family member to leisure places and other places. As mentioned earlier, some women participants viewed weekdays, including Monday, as their “free time” for socializing and spending time with friends due to family gatherings on weekends. This aspect may help to shape the temporal and coupling characteristics of travel for older women. For both genders, the travel distance for most leisure places was often less than 500 m when there were more than two companions, especially for activities such as playing with grandchildren in neighborhood gardens, as reported by some participants. In contrast, older men’s travel distances were notably more concentrated, as discussed in the previous section. They were often alone or accompanied by one family member, typically their spouse.

The choice of transportation was shaped not only by activity companions but also by the location of activities and travel distance. Walking and cycling emerged as the primary transportation modes, indicating that the local facilities in the case study area were well-suited to fulfilling the basic daily needs of the participants. However, insights from interviews revealed that some participants, particularly women, mentioned, “My son and daughter-in-law drove us for outings …”. This emphasizes that companionship from family members not only encouraged longer-distance leisure trips but also allowed older adults to make use of the transportation resources possessed by their companions. Moreover, one participant mentioned that she used to commute to work by bus before retirement, and even after retiring, she had retained the habit of taking the bus. This underscores that factors beyond activity characteristics, such as life experiences and habits, also play an important role in post-retirement travel behavior.

## 4. Discussion

The present study delved into the daily accompanied mobilities of older women and men in Tianjin, China, utilizing time-geographical activity diaries and semi-structured interviews. Our research reveals three key findings. Firstly, older women exhibit higher engagement with their families, attributable to both a greater percentage and extended duration of activities with family members. Secondly, despite older men participating in fewer activities at home, their travel distances remain more concentrated within a 1500 m radius compared to older women. Thirdly, older women demonstrate a tendency to engage in a broader spectrum of activities in diverse locations, including longer-distance leisure travel with family members, setting them apart from older men.

This study distinguishes itself by applying geographical theory to the well-established notion that women tend to play a more proactive role in their social interactions, as supported by numerous gerontological and psychological studies [43,45,46]. While prior geographical research on older adults’ social activities has been limited, Enßle et al. [47] emphasized the significance of spatial proximity in fostering neighborhood connections among older adults in Germany, particularly in times of weakened family ties. In the context of China, family ties remain highly important for older adults, especially amidst the challenges posed by COVID-19. Therefore, this study not only contributes to the existing body of literature on gender-related social interactions, but also introduces a geographical perspective to analyze the complexities of older adults’ social connectedness within the unique context of China.

Our findings highlight the diversity of activities that accompanying family members bring to older adults, particularly women. Although traditional gender roles, such as “men as breadwinners and women as homemakers”, continue to influence societal dynamics, they do not necessarily disadvantage Chinese older women in terms of their mobilities. While previous research has often emphasized the negative impacts of gender-related roles and family responsibilities on women’s mobility [24,48,49,50,51], we argue that these roles provide older women with valuable social support, enabling them to engage in a variety of activities in different places. For instance, caring for grandchildren is a common household responsibility for many older Chinese adults, particularly older women. This gender-related task involves a combination of domestic and outdoor activities, as our participants described taking their grandchildren to play in gardens and parks or accompanying them to dance classes using public transportation. This diversity in activities promotes a wide range of activity places and transportation choices. According to our interviews, active participation in family activities also allows older women to leverage their family’s transportation resources for longer-distance leisure travel. In contrast to the Western context, where mobilities related to religious activities [52] and exercising in gyms [53,54] are commonly reported among older adults, these types of activities were absent among our participants.

Furthermore, women’s earlier retirement age in China contributes to the additional time they spend with their families compared to men. In China, women retire at the age of 55, which is 5 years earlier than the retirement age for men. This is in contrast to the retirement age of 65 in most countries worldwide. While this retirement age difference reflects gender disparities and discrimination in the labor market [55], the value it brings to families when women retire earlier should not be underestimated. During our interviews with a married couple, in which the wife had already retired while the husband had not, the wife mentioned, “I retired earlier than him (her husband), so I get to spend more time playing with our grandson.” Many of the existing studies that use China as a case study may have chosen to adopt the international standard of 65 years for sample selection, possibly for the sake of international comparisons. However, we firmly believe that the unique context of women’s early retirement in China should not be overlooked.

In comparison to older women, older men tend to engage in fewer activities with family members and travel shorter distances. This pattern is not solely contributed by the availability of companions but is also attributed to the prevalent use of walking as the primary mode of transportation in the daily lives of older adults. This emphasis on walking among older adults is consistent with findings from other studies conducted in China [24]. In Western contexts, older women are often considered more vulnerable in terms of driving mobility when compared to men [13,18]. Although we did not specifically investigate factors such as vehicle ownership among our participants, there may not be substantial gender differences in this regard, as evident from their transportation preferences. Consequently, the characteristics of older men, who engage in fewer accompanied activities and rely on shorter-distance travel, become more pronounced under these circumstances.

Additionally, the impact of COVID-19 cannot be underestimated, particularly in explaining why our participants devoted more time to solitary activities in comparison to previous studies. McKenna et al. [56] conducted a study employing diaries to analyze the time allocation of older adults and reported that they typically spend around 42.00% of their time alone. It is important to note that our study involved participants with a mean age of 68.98 years, which is notably younger than the average age of 75 years in the McKenna et al. study. Despite this age difference, our findings reveal that both genders still allocate more than 50.00% of their time to solitary activities on both Sunday and Monday. This outcome can be attributed to the enduring anti-epidemic measures in place during our study period, coupled with the heightened vulnerability of older adults to infection and the psychological concerns expressed by our participants during interviews. Consequently, it is not surprising that our participants, despite their relatively younger mean age, spend an increased amount of time alone.

Future research could delve into the unique tradition of caregiving division in Tianjin. Specifically, in Tianjin, there exists a custom that places the responsibility of caring for grandchildren on maternal grandmothers. This is in contrast to the practice in most other northern cities where the paternal grandmother usually takes on this role, as indicated by our participants during interviews. This tradition explains two significant aspects: first, older women, rather than men, predominantly assume the responsibility of caring for grandchildren, and second, the patriarchal-centric family tradition may be challenged in Tianjin. While co-residence with sons, especially with a married or eldest son, is a strong preference in East Asia [57,58,59], including China, the custom in Tianjin deviates from this norm. It is worth further exploring whether this unique local culture and family relationship in Tianjin contributes to the travel behavior of older adults. For example, do maternal grandparents travel more frequently with their grandchildren than paternal grandparents in Tianjin? Moreover, caring for grandchildren by maternal parents may not necessarily be unique to Tianjin. Influenced by the “family planning” policy in the early stages of China’s development, the prevalence of only one child per family might have weakened the traditional preference for male descendants, encouraging older adults to care for their grandchildren, whether they are paternal grandparents or maternal grandparents. During the interviews, we did observe instances where grandmothers taking care of their grandchildren mentioned having only one daughter or son. It is unfortunate that we could not locate any existing research on this unique tradition or its influence on caregiving activities among older adults in Tianjin. Therefore, it is worthwhile for future studies to explore the intricate family relationships of older adults in Tianjin, specifically focusing on this distinctive caregiving tradition.

From a socio-spatial perspective, there are several practical recommendations. Firstly, given that most family-accompanied activities primarily occur on weekends, it would be valuable to promote flexible working hours for the workforce. This flexibility can enable them to spend more time accompanying older adults to promote their mobilities, thus alleviating the caregiving burden on older adults and contributing to the well-being of the entire society; specifically, allowing employees to decide their own working hours, based on ensuring the monthly total working time, to assist and accompany elderly family members when they go out. In addition, the post-pandemic era has promoted the implementation of online work, and a combination of online and offline work models can enable the workforce to work in virtual spaces while also caring for elderly family members in physical spaces. Secondly, the COVID-19 pandemic has led to a decrease in non-family-related activities, such as informal, non-organized activities such as square dancing, according to the interviews. This has highlighted the vulnerability of such activities during health crises. To address the reduced social interaction resulting from these crises or unexpected situations, it is recommended to increase the frequency of organized community activities, providing alternative avenues for social engagement and leisure among older adults. For example, older adults can apply to organize activities, and the community committee provides security, a place, medical, and health support to ensure social interactions during extraordinary periods.

There are several limitations to acknowledge in this study. Firstly, the small sample sizes for both men and women limited our ability to thoroughly explore population differences, making it challenging to dissect each gender group into subgroups based on demographic variables. Future research should prioritize larger sample sizes to enable more effective analysis of demographic factors. Secondly, to confirm how Tianjin’ local cultural context on caring for grandchildren shapes activities and mobilities of older adults, future research, in addition to requiring extensive questionnaire surveys, also needs more systematic interviews in terms of methods. Thirdly, the prevalence of COVID-19 and the strict anti-epidemic measures in place during the study period emphasized the significance of parks as primary outdoor recreational spaces for older adults. However, these circumstances constrained the scope of our surveys, preventing us from covering a broader geographic area and conducting more extensive and in-depth interviews over extended durations. Fourthly, due to the special social context, our findings may only be applicable to China or countries or regions with similar contexts. It is essential to explore how the coupling constraints of companions shape the daily mobilities of older adults in diverse social contexts and to conduct comparative studies, including different cities in China, other Asian countries, and Western regions. The cultural diversity in these contexts may contribute to various mobility patterns among older women. Moreover, recognizing that older adults navigate their daily mobilities within specific locations, it is imperative to compare the socio-spatial contexts between urban and rural settings to gain a more comprehensive understanding of the factors at play.

## 5. Conclusions

To explore the daily accompanied mobility patterns of older men and women, this study centered on the interaction between coupling constraints and factors such as travel distance, activity locations, and modes of transportation on both weekdays and weekends through the use of activity diaries and semi-structure interviews. The findings emphasize the pivotal role that the type, size and composition of activity companions play in influencing travel distances, the variety of activity places, and the choice of transportation modes, particularly for older women. These findings can be elucidated by considering older women’s enhanced social interactions from a spatiotemporal perspective, their earlier retirement compared to men in China, and the impact of COVID-19.

This study provides intriguing perspectives for comprehending the mobility of older adults by employing time–geographical methods and categorizing activity companions into various types and sizes, which have not garnered significant attention in geographical research. Consequently, this study underscores the significance of socio-spatial context, particularly the role of activity companions in shaping the mobility of older adults. Therefore, we emphasize the value of flexible work hours for the workforce and organized community-based activities. Furthermore, in comparison to related studies conducted in Western contexts, it challenges the prevailing notion that older women are more vulnerable in terms of mobility. Instead, it unveils the possibility of Chinese older women enjoying more advantageous mobilities, particularly in accompanied activities. Subsequent research endeavors should delve deeper into Tianjin’s local culture and customs regarding grandchild care, as well as accompanied mobilities in different social contexts.

## Figures and Tables

**Table 1:** Size of companions on Sunday and Monday for older adults.

Companion Type and Size	Sunday				Monday			
	Men		Women		Men		Women	
Family companions	*n*	%	*n*	%	*n*	%	*n*	%
0 (Alone)	901	53.25	703	50.04	932	54.28	713	50.11
1	609	35.99	534	38.01	637	37.10	574	40.34
2	55	3.25	66	4.70	55	3.20	41	2.88
3	28	1.65	25	1.78	16	0.93	23	1.62
4	25	1.48	25	1.78	16	0.93	17	1.19
5	3	0.18	2	0.14	3	0.17	0	0.00
Non-family companions								
1	64	3.78	64	3.78	56	3.26	41	2.88
2	2	0.12	2	0.12	2	0.12	10	0.70
Family and non-family								
2	5	0.30	2	0.14	0	0.00	4	0.28
Total	1692	100.0	1405	100.0	1717	100.0	1423	100.0

**Table 2:** Percentage of different types of places on Sunday and Monday.

	Home				Leisure Place			
	Sunday		Monday		Sunday		Monday	
	*n*	%	*n*	%	*n*	%	*n*	%
Men	1352	79.91	1381	80.48	251	14.83	236	13.75
Women	1175	83.63	1179	82.85	163	11.60	159	11.17
	**Service place**				**Others**			
	**Sunday**		**Monday**		**Sunday**		**Monday**	
	***n***	**%**	***n***	**%**	***n***	**%**	***n***	**%**
Men	62	3.66	72	4.20	27	1.60	27	1.57
Women	42	2.99	42	2.95	25	1.78	43	3.02

Note: *n* in this table represents the number of activity records.

## Data Availability

Data are contained within the article.
